# Quality of death notification forms in North West Bank/Palestine: a descriptive study

**DOI:** 10.1186/s13104-017-2469-0

**Published:** 2017-04-11

**Authors:** Jamal A. S. Qaddumi, Zaher Nazzal, Allam R. S. Yacoup, Mahmoud Mansour

**Affiliations:** 1 Faculty of Medicine and Health Sciences, An-Najah National University, PO box 7, Nablus, Palestine; 2 Department of anesthesia, An-Najah National University Hospital, Nablus, Palestine; 3 Department of general surgery, Palestine Medical Complex, Ramallah, Palestine

**Keywords:** Death notification form, Cause-of-death, Quality, Palestine

## Abstract

**Background:**

The death notification forms (DNFs) are important documents. Thus, inability to fill it properly by physicians will affect the national mortality report and, consequently, the evidence-based decision making. The errors in filling DNFs are common all over the world and are different in types and causes. We aimed to evaluate the quality of DNFs in terms of completeness and types of errors in the cause of death section.

**Methods:**

A descriptive study was conducted to review 2707 DNFs in North West Bank/Palestine during the year 2012 using data abstraction sheets. SPSS 17.0 was used to show the frequency of major and minor errors committed in filling the DNFs.

**Results:**

Surprisingly, only 1% of the examined DNFs had their cause of death section filled completely correct. The immediate cause of death was correctly identified in 5.9% of all DNFs and the underlying cause of death was correctly reported in 55.4% of them. The sequence was incorrect in 41.5% of the DNFs. The most frequently documented minor error was “Not writing Time intervals” error (97.0%).

**Conclusion:**

Almost all DNFs contained at least one minor or major error. This high percentage of errors may affect the mortality and morbidity statistics, public health research and the process of providing evidence for health policy. Training workshops on DNF completion for newly recruited employees and at the beginning of the residency program are recommended on a regular basis. As well, we recommend reviewing the national DNFs to simplify it and make it consistent with updated evidence-based guidelines and recommendation.

## Background

Death notification form (DNF) is a permanent legal record of the fact of death and without it no burial permit can be given [1]. Complete and accurate DNF is an important information source at both national and local levels. DNF are widely used, such as for the proof of death, computing mortality and morbidity statistics, developing health program and policy, and for medical and public health research [2–6].

In Palestine, the DNF is composed of two sections. (A) The demographic characteristics of the decedent section which includes full name of deceased, date of birth, gender, place of residence, marital status, time and place of death etc. (B) The cause-of-death (COD) section which consists of two parts. Part 1 in COD section is for reporting a chain of events leading directly to death, with the immediate COD (the final disease, injury, or complication directly causing death) and the underlying COD (the disease or injury that initiated the chain of morbid events that led directly and inevitably to death). Part 2 in COD section is for reporting all other significant diseases, conditions, or injuries that contributed to death, but did not result in the underlying COD reported in Part 1.

Errors in death notification are common [7], and worldwide [8]; range from incomplete notification, to inaccurate cause and manners of death [9], and using abbreviations [10]. One system divides the errors into major and minor [11] (for more details see Table 1).

**Table 1:** Types of errors in filling DNFs

Major errors	Minor errors
No underlying cause of death after mechanism	Absence of time intervals for each diagnosis
Incorrect sequencing of cause of death	Abbreviations used
Competing causes of death	Irrelevant information
No acceptable cause of death	Illegible handwriting

*DNFs* death notification forms

Up to our knowledge, no studies assessing the quality of death notification have been conducted in Palestine. A Pilot study was conducted in November 2011 in Nablus province by an author of this study and medical students at An-Najah National University in Palestine. The study revealed that errors were abundant and common. The aim of this study is to evaluate the quality of DNFs in terms of completeness and types of errors in the COD section in order to improve the quality of death certification in Palestine.

## Methods

### Study design and population

A descriptive study was conducted during the period of 1st September to 31st December 2013 at the primary health care directorates (PHD) at North West Bank (NWB)/Palestine to explore the errors of DNF and to assess the most common type of error. PHD is the central unit where all DNFs are reported directly from the peripheral level (i.e., hospitals and private Clinics).

The study population included all the DNFs during the year 2012 at the PHD in NWB/Palestine. The DNF is standard form distributed by the Palestinian Ministry of Health (PMoH) to all health care facilities and primary clinics in Palestine, and after filling, it’s sent back to the PHD to be evaluated and issued.

Sample size, based on expected proportion of 50, 95% confidence interval and 5% absolute precision on either side of the proportion, was 547 DNFs. Systematic random sampling technique was used. Every 5th DNF from the list of DNF documentation numbers in Vital Statistics Unit in PHD was included in the study sample.

### Data collection

Data abstraction sheet was constructed by the researchers based on national standards and international guidelines [1, 11] to collect information regarding the errors in filling the COD section in the DNF. Researchers reviewed the DNFs independently and any disagreements were resolved by consensus based on discussion.

Major errors included: (i) incorrect sequencing chain of events leading directly to death, (ii) competing causes of death occurs when two or more causally unrelated, etiologically specific diseases listed in part 1 of the DNF, (iii) unacceptable cause of death is documented when signs, symptoms or ill-defined terms such as old age or severe headache are listed in part 1 of the DNF. (iv) No underlying cause of death after mechanism; occurs if no underlying cause of death is reported and the notifying physician only uses mechanism of death or reported mechanism of death with incorrect underlying cause of death or there is no link between them. On the other hand, the minor errors include: absence of time intervals for each diagnosis, use of abbreviations, filling irrelevant information and illegible handwriting (see Table 1).

Furthermore, we collected information such as age, residency, gender, marital status, and notifying physician. The study protocol was approved by the Institutional Review Board (IRB) at An-Najah National University and by the PMoH. All collected data were treated confidentially.

### Statistical analysis

Statistical Package for the Social Sciences (SPSS) version 17.0 was used for data entry and analysis. Frequency tables, bar charts were used to describe study results. Chi square used for inferential part.

## Results

A total of 547 DNFs were evaluated in the present study. Nearly half (52.6%) were males, and three forth of them above 50 years old. The majority (61.2%) of DNFs were filled in hospitals. In 47.5% of the DNF, the certifiers were resident physicians compared to 6.8% were forensic physicians (for more details see Table 2).

**Table 2:** Characteristics of deceased persons in DNFs (N = 547)

Variable	Frequency (%)
Age (years)	
<1	32 (5.9%)
1–29	53 (9.7%)
30–49	51 (9.5%)
>50	410 (75%)
Residency	
City	155 (28.3%)
Village	346 (63.3%)
Camp	46 (8.4%)
Gender	
Male	288 (52.6%)
Female	259 (47.1%)
Location of death	
Governmental Hospital	264 (48.2%)
Non-governmental Hospital	71 (13.0%)
Home/clinic	212 (38.8%)
Notifying physician	
Resident	260 (47.5%)
Specialist	102 (18.6%)
Forensic	37 (6.8%)
General practitioners	148 (27.1%)

**Fig. 1:**
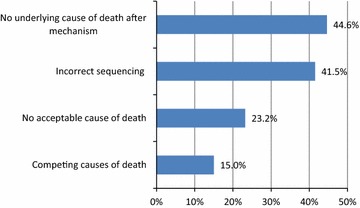
Frequency of major errors in death notification forms (N = 547). The frequencies of major errors made by physicians in filling the death notification form

The DNFs were evaluated for errors in filling the COD section. The majority (67%) of the DNFs had at least one major error. The most common major error was “No underlying cause of death after mechanism” (44.2%) followed by “Incorrect sequence” error (41.3%) (see Fig. 1).

**Fig. 2:**
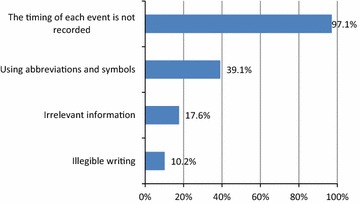
Distribution of minor errors in death notification forms (N = 547). The frequencies of minor errors made by physicians in filling the death notification form

When assessing DNFs for minor errors, only 12 (2.0%) DNFs were free of any minor error, and 535 (98.0%) had at least one minor error. The most frequently reported minor error was “Not writing Time intervals” error (97.0%) followed by “Using abbreviations and symbols” error (39.1%) (see Fig. 2).

The frequency of major errors in the DNFs was studied in relation to deceased and reporting physicians’ characteristics. Most of the major errors were committed equally between the different groups. DNFs written outside the hospital (home or clinic) had more (28.8%) “No acceptable cause of death” error than DNFs written in hospitals (20.3%) and that was statistically significant (P = 0.023) (see Table 3).

**Table 3:** Major errors in DNFs in relation to characteristic of deceased

Characteristics	Types of major errors					
	Incorrect sequence		No acceptable cause		Writing mechanism only	
	n (%)	P value*	n (%)	P value*	n (%)	P value*
Age of deceased (years)						
<1	11 (44.0)	0.731	10 (31.3)	0.379	14 (43.8)	0.649
1–29	20 (46.5)		12 (22.6)		20 (37.7)	
30–50	19 (48.7)		8 (15.4)		26 (50.0)	
>50	177 (52.7)		99 (24.1)		184 (45.0)	
Gender of deceased						
Male	123 (53.5)	0.350	60 (20.8)	0.062	131 (45.5)	0.618
Female	104 (48.6)		69 (26.4)		113 (43.8)	
Delay (days)						
<7	178 (52.2)	0.364	95 (22.2)	0.165	190 (44.5)	0.922
≥7	49 (47.1)		34 (28.3)		54 (45.0)	
Location						
Hospital	143 (51.6)	0.740	68 (20.3)	0.023	148 (44.2)	0.800
Home	84 (50.0)		61 (28.8)		96 (45.2)	
Hospital						
Governmental	112 (52.3)	0.662	53 (20.1) 15 (21.1)	0.845	117 (44.3)	0.921
	31 (49.2)				31 (43.7)	
Non-Governmental	31 (49.2%)		15 (21.1%)		31 (43.7%)	
Notifying physician						
Resident	111 (51.9)	0.340	52 (20.0)	0.266	112 (43.1)	0.605
Specialist	50 (56.8)		26 (25.5)		50 (49.0)	
Forensic	10 (38.5)		9 (24.3)		14 (37.8)	
G.P.	56 (47.9)		42 (28.4)		68 (45.9)	

*DNFs* death notification forms

* Chi squared test

## Discussion

Surprisingly, completely and correctly filled COD section was identified only in 1% of the DNFs. This result is consistent with a study in India where 1.2% of the DNFs had the COD section completed correctly. This shows a great lack of accuracy in filling the DNFs.

The immediate cause of death was identified correctly in 5.9% of evaluated DNFs. Most (92.7%) of the terms used in the line a (1st line in DNF) described mechanism of death like cardio-respiratory arrest, respiratory failure and heart failure, instead of writing the “immediate cause of death”. This is comparable (80%) with a study [12] conducted in Vadodara/India. It is surprising to find this high proportion of such error. Mechanism of death is a physiologic derangement or biochemical disturbance by which a COD exerts its lethal effect and should not be reported as the immediate COD. This situation was worsening by the fact that 44.7% of the DNFs contain the mechanism of death without reporting the underlying cause of death; the most common major error reported in our study. This is comparable (35%) with a study in Greece [13]. However, it was reported between 7 and 18% in other studies [14–16]. Physicians usually find it difficult to distinguish between the cause of death and the mechanism of death. They most often target their medical treatment to the mechanism [13]. Additionally, it is often not easy to identify the definite cause of death, especially in complex cases and those dying outside the hospitals. Reporting the mechanism of death without the underlying cause of death is a serious problem that can affects the quality of the DNF and limits its uses.

“Incorrect sequence” was the second most (41.5%) common major error in this study. This is similar to what have been reported in the study conducted in Vadodara [12]. In contrast, much less proportions (9, 24, and 28.7%) of this error have been found in Taiwan and South Africa [14–16]. This is also one of the major errors that limits the uses of the DNFs and makes it difficult to extract the underlying cause of death; the most important piece of information in the DNF.

“No acceptable cause of death” and “competing causes of death” were the third and fourth most (23.2 and 15%, respectively) common major errors in the DNFs. This is comparable with results of two studies in South Africa [15, 16] where 14.8 and 17.3% respectively of the reviewed DNFs had “No acceptable cause of death” error, and 15.3 and 14.9% respectively had “competing causes of death” [15, 16]. Increased life expectancy has led to a multivariate cause of death, and it is more and more likely that a deceased person have more than one disease such as cardiovascular disease, diabetes mellitus, cancer and others. As a result, the physicians may find it difficult to assign a single cause of death in such complex cases, particularly those dying outside the hospitals.

The majority (98%) of DNFs contained at least one minor error. That was consistent with the study in Vadodara which revealed that at least one minor error in all (100%) of the DNFs [12]. Similarly, the two studies conducted in South Africa in 2007 and in 2009 revealed that (91.7 and 98.4% respectively) of the reviewed DNFs had at least one minor error [15, 16].

The most common minor error (97%) was the “Absence of time interval”. The DNFs reviewed in the two studies conducted in South Africa in 2007 and in 2009 had “Absence of time interval “as the most common minor error (81.5, 98.4% respectively) [15, 16].This percentage is similar to a study result conducted in Vadodara (92.5%) [12]. Reporting the time estimate for each condition in the COD section is essential in providing complete picture of the cause of death and determining underlying cause of death. Physicians should pay attention to these entries as they give the chronology of events and provides a useful check on the accuracy of the reported sequence of conditions which can prevent major error of improper sequencing.

The second most common minor error was the use of abbreviation and symbols (39.1%). This is consistent with the study in Vadodara (32.5%) [12], and in contrast with the studies in South Africa in 2007 and in 2009 (23.7 and 10.7% respectively) [15, 16]. “Irrelevant information” and “illegible writing” were the third and fourth frequent (17.6 and 10.2%, respectively) minor errors and were consistent with Burger et al. study [15] which found that these errors were least frequent (13 and 2.5%, respectively) minor errors.

The majority of end users of DNF never went to medical school; this renders the form difficult to interpret and to extract the true cause of death related information. Although minor errors may not significantly affects statistics on the underlying cause of death, they should be avoided. In addition, these minor errors signify a degree of physicians’ inattention or lack of experience.

This study had some limitations. First; the fact that only the DNFs of North West Bank/Palestine were reviewed in the study may limit its generalisability. However, the results of the study showed that the three provinces included in the study had limited accuracy of DNF and death notification procedure with little differences in some types of errors. This may lead us to conclude that the inaccuracy in DNF may be generalized on whole Palestine as most physicians in Palestine have generally similar characteristics and background. Second; the evaluation of the “cause of death” section was researcher dependent; we tried to avoid this via evaluating each DNF by the researchers, independently.

## Conclusions

Almost all DNFs contained at least one minor or major error. This high percentage of errors may affect the mortality and morbidity statistics, public health research and the process of providing evidence for health policy. We recommend increasing the awareness of physicians about the importance of DNFs and its implications on improving their practice. This could be through offering training workshops on DNF completion for newly recruited employees and developing a manual on filling out the DNF with a clear instructions and guidelines. The DNFs are better to be reviewed for information accuracy by the attending physician and health care administrators prior to submission to higher authorities. Considering filling DNFs electronically will be helpful especially for eliminating legibility and abbreviation problems and for completion of any missing information once available. Thus, filling DNFs properly will improve accuracy of the national mortality report and, consequently, the evidence-based decision making.

## Abbreviations

COD: cause of death
DNFs: death notification forms
IRB: Institutional Review Board
PMoH: Palestinian Ministry of Health
NWB: North West Bank
PHD: Primary Health Directorate
SPSS: Statistical Package for the Social Sciences
